# Retrospective Screening for SARS-CoV-2 RNA in California, USA, Late 2019

**DOI:** 10.3201/eid2610.202296

**Published:** 2020-10

**Authors:** Catherine A. Hogan, Natasha Garamani, Malaya K. Sahoo, ChunHong Huang, James Zehnder, Benjamin A. Pinsky

**Affiliations:** Stanford University School of Medicine, Stanford, California, USA (C.A. Hogan, N. Garamani, M.K. Sahoo, C. Huang, J. Zehnder, B.A. Pinsky);; Stanford Health Care, Palo Alto, California, USA (C.A. Hogan, B.A. Pinsky)

**Keywords:** 2019 novel coronavirus disease, coronavirus disease, COVID-19, severe acute respiratory syndrome coronavirus 2, SARS-CoV-2, viruses, respiratory infections, zoonoses, diagnostic testing, screening, RNA, San Francisco Bay area, California, United States

## Abstract

To investigate the possibility of earlier cases of severe acute respiratory syndrome coronavirus 2 infection than previously recognized, we retrospectively tested pooled samples from 1,700 persons with respiratory signs/symptoms seen at Stanford Health Care, Palo Alto, California, USA, during the last 2 months of 2019. We found no evidence of earlier infection.

Phylogenetic analyses of severe acute respiratory syndrome coronavirus 2 (SARS-CoV-2) suggest virus emergence weeks, if not months, before the World Health Organization was notified of the original cluster of cases in Wuhan, China (*1*). These analyses have estimated that SARS-CoV-2 emerged from October 6, 2019, through December 11, 2019 (*2*). Given this timeline, interest in retrospectively identifying patient zero in different geographic areas has been growing, to better determine the spread of SARS-CoV-2 and to inform current and future surveillance strategies for emerging infectious diseases. 

Given the high volume of international travel before implementation of travel restrictions, travel-associated coronavirus disease (COVID-19) cases may have occurred in the United States earlier than previously recognized (*3*). However, monitoring for early community transmission of SARS-CoV-2 in the United States was challenging because the clinical manifestations of COVID-19 are similar to those of other respiratory virus infections, and emergence of COVID-19 overlapped with the annual respiratory virus season. In addition, local COVID-19 case finding and contact tracing efforts were limited by strict indications for testing based on specific risk factors, coupled with limited testing capacity (*4*,*5*). 

A case of COVID-19 in the San Francisco Bay area, California, was confirmed by autopsy on February 6, 2020. To determine whether the virus had been spreading earlier than previously recognized in northern California, we extended our recently reported pooled screening strategy (*4*) to a retrospective study that included the last 2 months of 2019.

Our study evaluated all nasopharyngeal swab samples collected October 31, 2019–December 31, 2019, at Stanford Health Care (Palo Alto, California, USA) for which sufficient residual sample volume was available. These samples were collected from inpatients and outpatients who had had negative routine respiratory virus test results (Respiratory Pathogen Panel; GenMark Diagnostics, https://www.genmarkdx.com, or Xpert Xpress Flu RSV; Cepheid, https://www.cepheid.com) and had not been tested for SARS-CoV-2. Pool size was determined after literature review, accounting for an expected prevalence of <1% (*6**,**7*). Pools were created by combining 10 nasopharyngeal samples, and screening was performed by real-time reverse transcription PCR targeting the nucleocapsid gene (region N2) (*8*). We extracted demographic characteristics for a randomly selected subset of 100 persons. Trends of routine respiratory virus positivity were examined for the same period covered by the retrospective SARS-CoV-2 testing. This study was approved by the Stanford institutional review board, and individual patient consent was waived.

We tested 1,700 individual nasopharyngeal specimens (170 pools) for SARS-CoV-2. Of these, 841 samples had previously tested negative by the Respiratory Pathogen Panel and 859 by the Xpert Xpress Flu RSV. From the subset of persons for whom demographic data had been analyzed, most (67%) were adults. Most (64%) persons had consulted the emergency department for testing, followed by an outpatient clinic (23%) or an inpatient ward (13%). No SARS-CoV-2–positive pools were identified. The study period corresponded to the onset of the 2019–2020 respiratory virus season, during which the number of cases of influenza A, influenza B, and respiratory syncytial virus increased and the frequency of other seasonal viruses varied (Appendix Figure) according to testing of separate samples collected during the same period as the study.

Pooled testing of 1,700 nasopharyngeal samples collected from persons in Palo Alto, California, who had respiratory signs/symptoms during the last 2 months of 2019 detected no case of COVID-19. This study and previous studies indicate that in the San Francisco Bay area, symptomatic persons without risk factors and with a SARS-CoV-2 infection diagnosis began seeking medical attention at the end of February 2020 (*9*,*10*).

Our study is limited by sampling from a single institution, corresponding to a population that may not be representative of the underlying area as a whole. Further retrospective SARS-CoV-2 reverse transcription PCR screening using specimens collected at other institutions throughout the United States will be needed to fully determine early community transmission. Our research will complement phylogenetic viral sequence analysis and large-scale seroprevalence studies to characterize the regional and national emergence of SARS-CoV-2. It is possible that use of pooled testing led to lower sensitivity; however, pool sizes of 10 samples maintain high performance compared with individual samples. Given that we included only samples negative for conventional respiratory viruses, we cannot exclude the possibility that we missed cases of SARS-CoV-2 co-infection with another respiratory virus.

Our pooled screening strategy for investigating local community transmission of SARS-CoV-2 in the San Francisco Bay area of California during late 2019 during the onset of the respiratory virus season identified no COVID-19 cases. This finding is consistent with limited transmission in this population at this time.

Appendix Respiratory surveillance, November and December 2019, California, USA, 2019–2020.
