# Supplementary material for: Retrospective Screening for SARS-CoV-2 RNA in California, USA, Late 2019
Source: Emerg Infect Dis. 2020 Oct;26(10):2487–8. doi: 10.3201/eid2610.202296 (PMC7510744; doi:10.3201/eid2610.202296)
Supplement: Appendix — Respiratory surveillance, November and December 2019, California, USA, 2019–2020. [file 20-2296-Techapp-s1.pdf]

Article DOI: https://doi.org/10.3201/eid2610.202296

Retrospective Screening for SARS-CoV-2 RNA in California, USA, Late 2019

Appendix

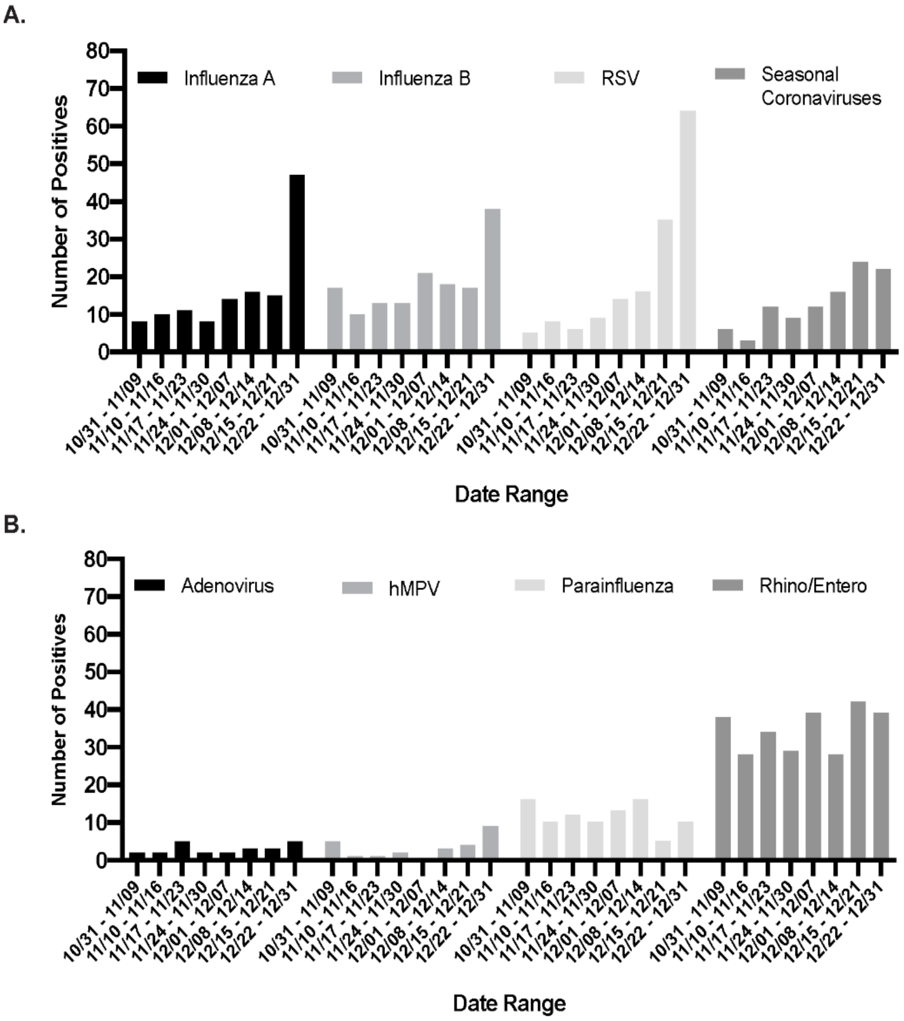

Appendix Figure. Respiratory surveillance November and December 2019. A) Influenza A, influenza B, respiratory syncytial virus (RSV), and seasonal coronaviruses, including HKU1, OC43, NL63, 229E. B) Adenovirus, human metapneumovirus (hMPV), parainfluenza viruses 1, 2, 3, and 4, and rhinovirus/enterovirus.
